# What are the top 10 global research priorities for perianal Crohn’s disease? The Global Perianal Crohn’s Disease Priority Setting Partnership

**DOI:** 10.1093/ecco-jcc/jjag018

**Published:** 2026-05-01

**Authors:** Theo Pelly, Easan Anand, Jonathan Gower, Sameer Mannick, Tom Hough, Sarah Markham, Shaji Sebastian, Christianne Buskens, Jeffrey D Mccurdy, Parakkal Deepak, Amir Reza Radmard, Jaap Stoker, Philip Lung, Paulo Gustavo Kotze, Lisa Younge, Denise Robinson, Shivani Joshi, Eathar Shakweh, Luke Hanna, Harjeet Singh, Yarunessa Khan, Katie Nightingale, Nick Powell, Phil Tozer, Ailsa Hart, PSP Collaborators, John Nik Ding, Sulak Anandabaskaran, Jeroen Geldof, Sara El Ouali, Melanie Seale, Angela Forbes, Kelly Exner, Tito Reinaldo, Rachel Capstick, David Bourada, Harsha Nagaraj, Kera Berutti, Anna Antoniou, Amber Harris, Devesh Varma, Sophie Verstraete, Tina Aswani-Omprakash, Vishal Sharma, Raihaneh Bokharai

**Affiliations:** St Mark’s Hospital and Academic Institute, London, United Kingdom; Imperial College London, London, United Kingdom; St Mark’s Hospital and Academic Institute, London, United Kingdom; Imperial College London, London, United Kingdom; James Lind Alliance, United Kingdom; Patient representative, United Kingdom; Patient representative, United Kingdom; Patient representative, United Kingdom; Hull University Teaching Hospital, Hull, United Kingdom; Department of Surgery, Amsterdam UMC, University of Amsterdam, Amsterdam, The Netherlands; University of Ottawa, Ottawa, Canada; Washington University in St Louis, St. Louis, MO, United States; Department of Radiology, Shariati Hospital, Tehran University of Medical Sciences, Tehran, Iran; Department of Radiology and Nuclear Medicine, Amsterdam UMC, University of Amsterdam, Amsterdam, The Netherlands; Amsterdam Gastroenterology, Endocrinology, Metabolism, Amsterdam, the Netherlands; St Mark’s Hospital and Academic Institute, London, United Kingdom; Imperial College London, London, United Kingdom; Colorectal Surgery Unit, Pontifícia Universidade Católica do Paraná, Curitiba, Brazil; St Mark’s Hospital and Academic Institute, London, United Kingdom; St Mark’s Hospital and Academic Institute, London, United Kingdom; St Mark’s Hospital and Academic Institute, London, United Kingdom; Imperial College London, London, United Kingdom; St Mark’s Hospital and Academic Institute, London, United Kingdom; Imperial College London, London, United Kingdom; St Mark’s Hospital and Academic Institute, London, United Kingdom; Imperial College London, London, United Kingdom; Post-graduate Institute of Medical Education and Research, Chandigarh, India; Crohn’s and Colitis, United Kingdom; Crohn’s and Colitis, United Kingdom; Division of Digestive Diseases, Faculty of Medicine, Imperial College London, London, United Kingdom; St Mark’s Hospital and Academic Institute, London, United Kingdom; St Mark’s Hospital and Academic Institute, London, United Kingdom; Division of Digestive Diseases, Faculty of Medicine, Imperial College London, London, United Kingdom

**Keywords:** Crohn’s disease

## Abstract

**Background and aims:**

Perianal Crohn’s disease (pCD) is a debilitating condition with a prevalence of approximately 20% in patients within 10 years of diagnosis of CD. The condition significantly impacts patient quality of life, and there remain a number of unmet needs relating to its optimal management. A global Priority Setting Partnership (PSP) was established to identify the top 10 research priorities for pCD.

**Methods:**

The PSP followed standard James Lind Alliance (JLA) methodology. An initial survey was circulated to gather unanswered research questions from key stakeholders. From these responses, a longlist of summary questions was formed. These questions were ranked by stakeholders in a second prioritization survey. The highest ranking 19 questions were taken into the final workshop, where a panel of people with lived or professional experience of pCD identified the top 10 research priorities.

**Results:**

Over 1200 individual research questions were submitted by over 400 respondents, from over 30 countries. From the 51 summary questions that were developed from these responses, the top 10 were identified in the final workshop. These included identifying the optimal combined treatment strategy across the full spectrum of pCD, reducing the impact on quality of life, improving timely diagnosis of pCD, identifying a biological classification of pCD, and understanding and managing the impacts on sexual function, intimacy, and relationships.

**Conclusions:**

This global PSP represents a major step towards stakeholder-driven and focused research in pCD. The research priorities provide a roadmap for researchers, funders and policy-makers to align future research with patient and clinician needs to improve outcomes.

## Graphical Abstract

**Graphical Abstract:**
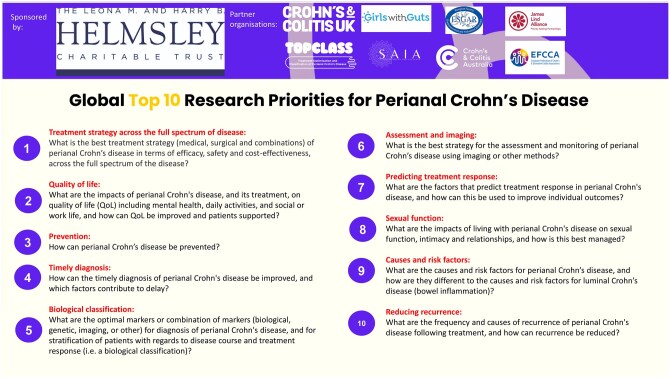

## 1. Introduction

Perianal Crohn’s disease (pCD) is a severe and complex manifestation of Crohn’s disease (CD), an immune-mediated inflammatory disease of the gastrointestinal tract characterized by relapsing and remitting transmural inflammation. pCD is associated with a prevalence of 20% within 10 years of diagnosis, and may manifest without, before, or after the onset of luminal inflammation.^1^^,^^2^ It includes diverse perianal pathologies such as fistulae, abscess, skin tags, anorectal strictures, and erosive disease such as fissures and ulcers,^3^ and results in symptoms such as anorectal pain, discharge, and incontinence. These symptoms culminate in diminished quality of life, challenges with intimacy, fatigue, and work impairment.^4^ The management of pCD is multi-disciplinary, involving combined medical and surgical treatment approaches and specialist nursing expertise.

The James Lind Alliance (JLA), a non-profit organization funded by the National Institute for Health and Care Research, was established in the UK in 2004. It aims to bring clinicians, patients, and researchers together to identify key areas of uncertainty for future research to address.^5^ “Evidence Uncertainties” in this context are defined as “questions about healthcare that cannot be answered by existing research.”^5^ Thus far, the JLA has facilitated the completion of over 150 Priority Setting Partnerships (PSPs) across a broad range of clinical topics.^5^

The 2017 JLA Inflammatory Bowel Disease (IBD) Priority Setting Partnership identified that determining an “optimal treatment strategy for perianal Crohn’s disease” was the fifth most important priority for future research in IBD.^6^ Despite this, progress in this CD phenotype remains suboptimal with poor understanding of etiopathology, limited options in therapy, and overall poor healing rates. Several research gaps persist, including understanding the pathogenic mechanisms underpinning fistulizing pCD and the role of microbiological, genetic, and immunological factors.^7^ Furthermore, clinical trials evaluating outcome measures relevant to patients with pCD remain scarce, resulting in a limited evidence base to inform both clinicians and patients regarding the efficacy of IBD therapies in this context. Despite advances in medical and surgical treatment approaches, high healthcare utilization and high rates of treatment failure remain significant challenges.^8^ The most effective strategy for combining or sequencing the available medical and surgical interventions is yet to be determined.

Until now, there has been no pCD-specific research prioritization exercises performed by the JLA or other organizations. Given the growing understanding of IBD as a global phenomenon, the impact of pCD may also increase, highlighting the need for a coordinated and inclusive research agenda. The Global Perianal Crohn’s Disease Priority Setting Partnership was therefore established to unify patients, clinicians, and researchers across borders to develop the Global Top 10 Research Priorities in Perianal Crohn’s disease.

## 2. Materials and methods

### 2.1. Study overview

**Figure 1.**
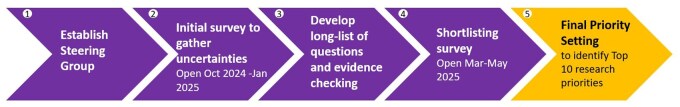
Overview of the process.

The Global Perianal Crohn’s disease Priority Setting Partnership in Perianal Crohn’s Disease (pCD PSP) was established in August 2024 in collaboration with the JLA. The project was carried out in accordance with the JLA methods, which are described in detail in the JLA guidebook (Version 10).^9^ An outline of the process and key dates for the pCD PSP is outlined in Figure 1. A steering group was established with 19 members from seven countries, across four continents including Europe, Asia, and North and South America. The steering group was purposively selected by the PSP co-leads (AH and PT). The steering group was multidisciplinary, including members with expertise in colorectal surgery, gastroenterology, radiology, specialist nursing, as well as patient and voluntary organization representatives, and a JLA advisor to facilitate the process. Full details of the steering group are listed in Appendix S3. The steering group met on average monthly during the project, and managed all aspects, including setting the scope and objectives of the pCD PSP, survey design, analysis, and dissemination of the findings. The work was supplemented by a data analysis subgroup (EA, TP, LH, SJ, ES) who led on the data analysis and evidence checking processes. Ethical approval was not required for this JLA PSP.

A number of global partner organizations were contacted to assist in recruiting survey respondents and promoting and disseminating the findings of the PSP. These included patient support and professional organizations. Participating partner organizations included the South Asian IBD Alliance, United European Gastroenterology, Crohn’s and Colitis UK, The Treatment Optimisation and Classification of Perianal Crohn’s Disease (TOpClass) consortium, The European Society of Gastrointestinal and Abdominal Radiology, Girl with Guts, Crohn’s and Colitis Australia, Dukes’ Club, European Federation of Crohn’s and Colitis Associations, and the Crohn’s and Colitis Foundation (America). Details of the partner organizations that agreed to assist in this project are listed in Appendix S3.

The aims of the global pCD PSP were to identify uncertainties relating to pCD across all aspects of the patient pathway. The scope was defined to include all uncertainties including etiology, risk factors, definitions and classifications, diagnosis, investigations and treatments, postsurgical concerns, and all aspects of the patient pathway, as outlined in the PSP protocol.^10^ Uncertainties relating to both fistulizing and non-fistulizing conditions, and associated conditions such as anorectal carcinoma and hemorrhoids, were also considered within scope. Questions from or relating to both adult and pediatric populations were included. Questions relating to the development of pCD due to monogenic disorders and those relating to hyperlocalized regional infrastructure issues were considered out of scope.

### 2.2. Initial uncertainty collecting survey

The electronic survey software Qualtrics (Provo, UT, USA) was used to generate an initial survey, allowing respondents to freely list three uncertainties relating to pCD. Basic demographics were collected, including whether the respondent was a patient or clinician, age, geographic location, specialty, area of expertise, and work settings. To ensure global reach and representation, the survey was translated into eight languages, including Arabic, Simplified Chinese, Dutch, French, Hindi, Portuguese, Spanish, Turkish, Swahili, and Urdu, by a professional translation service (Absolute Translations Ltd, London, UK). The survey content, phrasing, and usability were tested by patient representatives in the steering group. The survey was advertised and circulated via professional and patient networks of the steering group members, partner organization mailing lists, and a promotional campaign on various social medica channels. The initial survey was also promoted at the UEG (United European Gastroenterology) conference in 2024 via a poster presentation and at the TOpClass consortium meeting at the same conference.^11^ Survey responses were processed by the data analysis team. Out-of-scope responses were removed. In-scope responses were coded to identify whether the respondent was a patient, carer, parent, healthcare professional (including sub-specialty), or volunteer. An additional identifier code was included for the country of the respondent. Themes were identified in the responses relating to all aspects of the patient pathway. Initial coding was conducted by TP and reviewed by the steering group. Responses were then grouped by theme into summary questions. This was initially performed by TP and EA, and then reviewed by the steering group. The summary questions were reviewed and developed through an iterative process until consensus was reached. The initial survey was open from October 14, 2024 to February 28, 2025, including a 4-week extension to ensure data saturation had occurred.

### 2.3. Evidence checking process

The summary questions were amalgamated into a spreadsheet. Each summary question was checked against the literature in a pre-defined search strategy outlined in the project protocol.^12^ This was performed by the data analysis team (TP/EA/LH/SJ/ES), to identify high-level (where available) and high-quality evidence including meta-analysis and systematic reviews. Evidence sources included MEDLINE, EMBASE, Cochrane Central database, CINAHL, Google Scholar, and NICE guidance. For each summary question, specific search terms were developed. The search was limited to results within the last 5 years (consistent with JLA methodology), with only English language and full-text publications included. The question verification process was completed on March 14, 2025, and each summary question was graded as “answered,” “partially answered,” or “unanswered.” The results of the evidence search were reviewed by the steering group.

### 2.4. Interim priority setting survey

Following the evidence checking process, the longlist of unanswered and partially answered research questions was developed into a second online survey using Qualtrics. Respondents were asked to select their 10 most important summary questions. The order in which the categories of questions were presented was randomized for each respondent. The survey was available in the same eight languages as the initial survey, and distributed via partner organizations as well as a mailing list created from initial survey respondents who had given consent to be contacted. Rankings were calculated for healthcare professionals and for patients, carers, or parents of those with lived experience of pCD. These rankings were used to generate the shortlist of 19 summary questions to take to the final workshop, in accordance with JLA methodology (see Appendix S2). The survey was open for 2 months, from March 21 to May 14, 2025.

### 2.5. Final workshop

The final workshop was a virtual event held over two days on June 2–3, 2025. Respondents were identified from those expressing an interest in the initial and interim surveys, as well as through professional networks of the steering group members and partner organizations, and were invited selectively to ensure demographic balance in terms of gender, location, and professional or lived experience. Fifteen patients attended the workshop, as well as two specialist nurses, two radiologists, seven gastroenterologists, and one colorectal surgeon. The workshop was facilitated by several JLA advisors using nominal group technique methodology. The participants were divided into small groups with mixed demographics by the JLA facilitators, in order to discuss the questions and rank the final 19 summary questions. These rankings were then combined, and on the second day the final rankings were again discussed, reconfigured, and combined to produce the final ranking of the 19 summary questions.

## 3. Results

The initial survey had a total of 464 respondents, generating 1200 individual research uncertainties. Respondents were from 30 countries across six continents, with the majority of responses from the UK (19%), USA (11%), The Netherlands (14%), Portugal (13%), and Brazil (9%). Patients provided 58% of responses. The majority of the responses were from high-income countries according to the World Bank criteria (80%) with 7% from upper middle-income countries and 13% from lower middle-income countries. The full demographics are listed in Table 1. Following thematic analysis of these responses, a total of 51 individual summary questions were produced.

**Table 1.** Demographics for the initial and interim survey.

		No.	%
**Initial survey**			
**Number of responses by country (top 10)**	UK	232	19.3%
	USA	134	11.2%
	The Netherlands	170	14.2%
	Portugal	151	12.6%
	Brazil	104	8.7%
	Iran	77	6.4%
	Belgium	68	5.6%
	Australia	43	3.6%
	Canada	35	2.9%
	Cyprus	21	1.8%
**Number of responses by respondent type**	Patient	697	58.1%
	Gastroenterologist	178	14.8%
	Surgeon	112	9.3%
	Radiologist	76	6.3%
	Specialist Nurse	37	3.1%
	Parent/Carer	51	4.3%
	Other	23	1.9%
	Psychologist	9	0.8%
	Dietitian	8	0.7%
	Pathologist	3	0.3%
	Volunteer	7	0.6%
**Number of responses by World Bank Income status**	High income	967	79.9%
	Upper middle income	82	6.8%
	Lower middle income	161	13.4%
**Interim survey**			
**Number of responses by country (top 10)**	UK	80	26.1%
	Australia	60	19.5%
	USA	41	13.4%
	Netherlands	29	9.4%
	Iran	18	5.9%
	India	15	4.9%
	Belgium	12	3.9%
	Italy	6	2.0%
	Portugal	6	2.0%
	Spain	4	1.3%
**Number of responses by respondent type**	A healthcare professional working with people with perianal Crohn’s disease	106	34.5%
	A parent or carer for someone with perianal Crohn’s disease	14	4.5%
	Other (please specify)	7	2.3%
	Person with perianal Crohn’s disease (currently or previously)	175	57.0%
	Voluntary organization representative	5	1.6%
	Volunteer or work as part of a group helping those with perianal Crohn’s disease	1	0.30%
**Number of responses by World Bank Income status**	High income	262	85.3%
	Upper middle income	26	8.5%
	Lower middle income	19	6.2%

The interim prioritization survey received 307 responses (Table 1). Responses were received from 30 countries globally, with most of the responses from the UK (26%), Australia (20%), USA (13%), the Netherlands (10%), and Iran (6%). Analysis of the results involved calculating the highest-ranking priorities separately for healthcare professionals in comparison to a combined group of patients, carers, parents, volunteers, and other respondents. These rankings were compared in accordance with the JLA methodology to produce the shortlist of 19 interim priorities to take forward to the final workshop.

**Figure 2.**
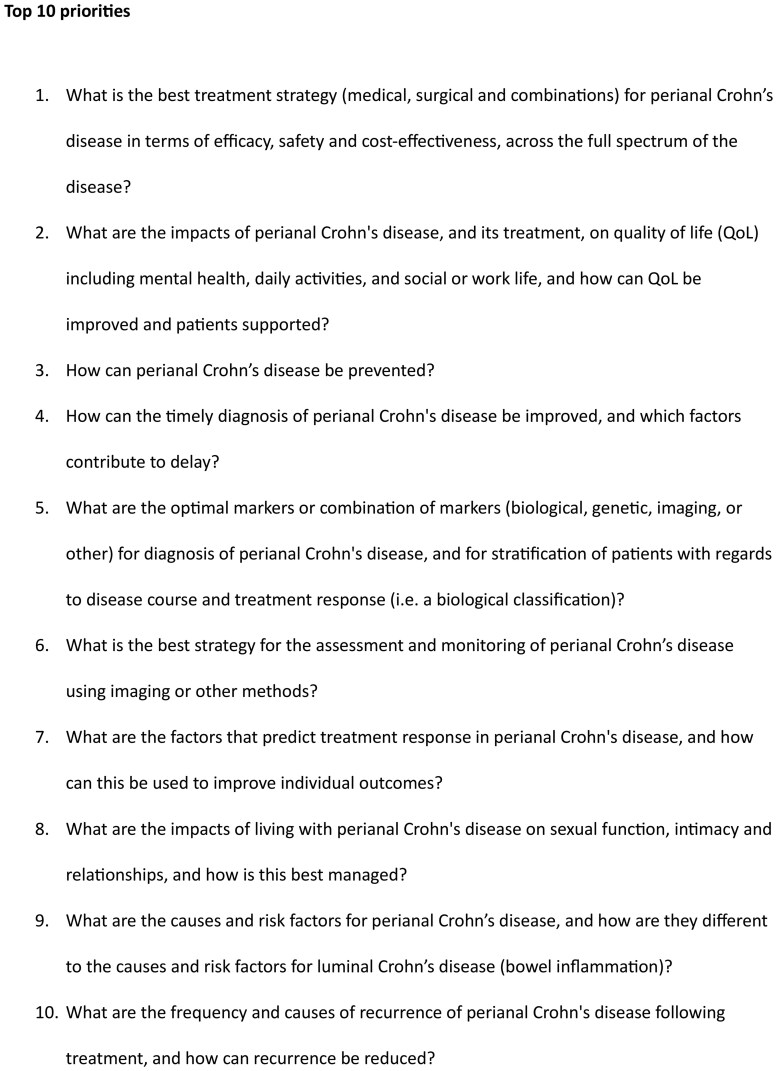

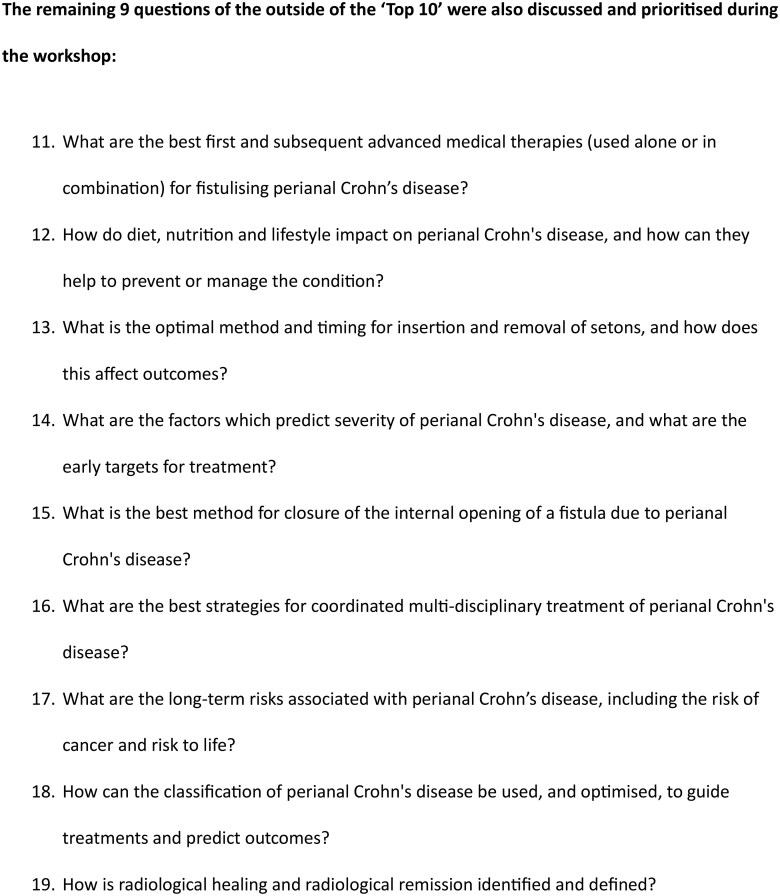
The global top 10 research priorities for perianal Crohn’s disease.

The final workshop was held as an online videoconference (Zoom, Zoom Communications, San Jose, Ca, USA) on June 2-3. A total of 27 participants attended the workshop. This consisted of 15 patients with lived experience of pCD (one of whom was also a general practitioner) and 12 healthcare professionals, consisting of seven gastroenterologists, two specialist nurses, one colorectal surgeon, and two radiologists. The geographic location of the participants and associated World Bank Income status is available in Appendix S3. The final Top 10 list of research priorities for pCD is listed in Figure 2, along with the full shortlist of 19 priorities that were taken into the Final Workshop.

## 4. Discussion

The global pCD PSP has successfully conducted an international research prioritization exercise to identify the top priorities for future research into pCD, in collaboration with the JLA. This is, to our knowledge, the first global priority setting partnership focusing on pCD. Input into the partnership was obtained through engaging with stakeholders, including patients, carers, healthcare professionals, researchers, and volunteers with experience of pCD.

This PSP expands upon the 2017 Inflammatory Bowel Disease Priority Setting Partnership, in which the fifth most important priority related to the best management strategy for pCD, and identifying the individual factors that determine this. In the pCD PSP, a number of novel and key themes were identified that advance on this, whilst also extending into new concepts. The highest-ranked priority was the identification of the optimal treatment strategy encompassing medical, surgical, and other therapies across the full spectrum of the disease. Currently, joint management with gastroenterologists and colorectal surgeons is the reference standard for the treatment of complex pCD. However, there is insufficient evidence to support clinical guidelines relating to the combination or sequential use of medical and surgical therapies in pCD.^13^ Additionally, the treatment of non-fistulizing pCD such as strictures and erosive disease remain an unmet need.^14^ Future research in pCD should focus on the development of an optimal treatment strategy across the “full spectrum” of the disease. This could include reducing recurrence rates following established therapies (10th priority).

Understanding the impact of pCD on quality of life (QoL) was recognized as the 2nd most important priority. Although disease-specific and partially validated QoL measures such as the Crohn’s Anal Fistula Quality of Life Index (CAF-QoL) have been developed, these are not used routinely in clinical research (or in clinical practice) with the majority of studies using generic QoL measures.^15^ There is a need to assess the impact of both existing and novel treatments on QoL, with the ultimate aim of improving outcomes in this population, and assess changes in QoL across the different classes of pCD.^15^^,^^16^ This was also highlighted in the 8th priority, which identified a need to further understand the impact on pCD on sexual function, relationships, and intimacy.

Another key theme was the prevention of pCD. Identifying the underlying etiological mechanisms and risk factors for pCD (the 9th priority) will allow researchers to develop effective prevention strategies and reduce the burden of pCD on patients, carers, and healthcare systems. The TOpClass classification for fistulizing pCD represents a practical and patient-centered system for categorizing patients as they move through discrete stages in the natural history of the disease. The 5th priority highlights a need to complement this classification with biological multiomics data and advanced and/or quantitative methods such as radiomics, to assist in diagnosis and prognostication. The importance of personalized medicine is well recognized in CD, and there is a need to improve predictive tools that identify non-response before treatments are commenced.^17^ This need is also acknowledged in the 7th priority, which highlights the importance of investigating factors that predict treatment response to improve outcomes for individual patients.

The 6th top priority relates to the assessment and monitoring of pCD. Whilst pelvic magnetic resonance imaging (MRI) is currently the reference standard investigation, the role of MRI indices, standardized definitions, and advanced MRI techniques such as quantitative MRI, volumetric analysis, 3D modeling, radiomics and machine learning require further research.^18^^,^^19^

### 4.1. Strengths

This PSP has several advantages. It addresses an important yet under-researched condition that has a long-lasting and significant impact on people with lived experience, setting the top 10 priorities for future research initiatives. It has been co-produced with all key stakeholders, including a range of healthcare professionals and patient representatives from across the globe.

Additionally, it draws on the strengths of the JLA’s standardized methodology, which yield a number of benefits beyond research funding, including establishing patient–professional relationships and strengthening global networks.^20^ During the process of this PSP, a number of patient and professional partner organizations based in different geographic regions successfully collaborated on delivering its objectives. A number of the priorities, for example those relating to the impact of pCD on daily life, work, relationships, and intimacy, as well the impact of diet and nutrition, are suitable early targets for future collaborative efforts by these organizations and others, to address these unmet needs. Increasingly, funding organizations recognize the JLA approach to involve stakeholders in agenda-setting for research. As such, the priorities identified in this PSP may support researchers globally to secure funding relating to pCD and collaborate with global networks when doing so.

### 4.2. Limitations

As with many priority-setting exercises, the scope of participation was inherently constrained by the demographics and availability of the key stakeholders that were able to engage in the process. This PSP aligns with several prior PSPs that have been completed with global representation. These include the PSP for Major Trauma, and the PSP for Burns Care.^21^^,^^22^ During the steering group selection process, efforts were made to involve steering group members with multidisciplinary experience from a range of different geographic regions, and although six countries are represented, the majority of steering group members were based in the UK. During the survey distribution and final workshop phases, despite efforts to contact and involve a number of patient and professional IBD organizations and involve professional networks, some geographic regions and low- and middle-income countries were less well represented. In particular, the responses from low- and middle-income countries in this PSP represented 20% for the initial survey, a factor that may have limited the representation of global views. pCD is relatively rare and has been historically less diverse in its prevalence in many low- and middle-income countries, and it is possible that this contributed to the difficulties in establishing relationships with stakeholders in these regions to contribute to the PSP. Future global PSPs should not underestimate this challenge, and perhaps distribute additional resources to establishing partnerships in low- and middle-income regions, in order to both increase contribution to planned PSPs and also provide future benefits beyond this. Of note, the Global Burns PSP had considerable success in utilizing grass-roots volunteers to improve engagement in low- and middle-income countries, achieving a proportion of 36% of survey respondents from low- and middle-income countries.^21^

Recent evidence suggests that IBD (including CD), which was traditionally considered to be rare in regions in Central and South America, is increasing in incidence in these areas.^23^^,^^24^ It is likely that there will be a resultant proportionate increase in pCD cases, which may increase the numbers of patients with pCD who can respond to future PSPs, although geographic variations in the clinical phenotypes of CD, including pCD, remain poorly understood. As these epidemiologic shifts occur, it will remain important to involve stakeholders from these regions in future research. The inclusion of perspectives from low- and middle-income countries in all stages of this PSP provides a foundation for broader applicability of the identified “Top 10” research priorities for pCD. Patients with pCD often encounter long delays to initiation of treatment.^25^ Acknowledging the paucity of evidence, it is reasonable to suggest that global regions, where pCD incidence is rising, may face greater delays due to limited awareness and diagnostic capacity. This challenge was recognized as the 4th top priority.

Routinely, the JLA seeks feedback on the final PSP workshop from all participants. Participants highlighted the empowering nature of the process, as well as the importance of listening to multiple different perspectives. Some attendees (in particular participants with lived experience of pCD) expressed disappointment that a question on diet and nutrition narrowly missed a place in the Top 10 (ranked 12th). It should be highlighted that this remains a priority for future research, and this is consistent with the top 10 research priorities in IBD, which included a question relating to the role of diet in the management of ulcerative colitis and CD as the third most important priority.^6^

### 4.3. Conclusions

The pCD PSP represents a significant international initiative to identify the most important research priorities for this complex condition. Using a collaborative approach based on JLA methodology, the PSP has brought together a diverse, global community of patients, carers, healthcare professionals, and researchers to identify the top 10 unanswered research questions in pCD. These priorities highlight critical unmet needs across the full spectrum of the disease, including the development of optimal treatment strategies, improved understanding of QoL impacts, and the need for prevention, personalized care, and better diagnostic and monitoring tools. The prioritization of these research areas illustrates the importance of multidisciplinary care and the integration of patient perspectives into research agendas. The response rate and involvement from low- and middle-income countries was low in the survey phases and final workshop, which may have limited the representation of global views. However, this initiative lays a foundation for more inclusive global collaboration as IBD epidemiology evolves worldwide. Moving forward, these research priorities offer a roadmap for investigators, funders, and policy-makers to address the most pressing gaps in evidence, with the ultimate aim of improving outcomes and care for people living with pCD globally.

## Supplementary Material

jjag018_Supplementary_Data

## Data Availability

All data will be available for publication on the James Lind Alliance website. This will include all deidentified participant data. The study protocol and terms of reference are published on the James Lind Alliance website. This information will be publicly available without restriction.
