# Supplementary material for: What are the top 10 global research priorities for perianal Crohn’s disease? The Global Perianal Crohn’s Disease Priority Setting Partnership
Source: J Crohns Colitis. 2026 May 1;20(4):jjag018. doi: 10.1093/ecco-jcc/jjag018 (PMC13134380; doi:10.1093/ecco-jcc/jjag018)
Supplement: jjag018_Supplementary_Data [file jjag018_supplementary_data.docx]

**Appendix**

**Appendix 1.**

Table showing the ranking of the top 19 priorities included in the final workshop, categorized by healthcare professionals, non-healthcare professionals, and overall combined rank.

| **Priority** | **Healthcare professional Rank** | **Non Healthcare professional Rank** | **Combined rank** |
| --- | --- | --- | --- |
| **What are the factors that predict treatment response in perianal Crohn's disease, and how can this be used to improve individual outcomes?** | **6** | **30** | **14** |
| **How can the classification of perianal Crohn's disease be used, and optimised, to guide treatments and predict outcomes?** | **8** | **15** | **7** |
| **How can the timely diagnosis of perianal Crohn's disease be improved, and which factors contribute to delay?** | **13** | **12** | **13** |
| **What are the optimal markers or combination of markers (biological, genetic, imaging, or other) for diagnosis of perianal Crohn's disease, and for stratification of patients with regards to disease course and treatment response (i.e. a biological classification)?** | **10** | **32** | **18** |
| **What is the optimal method and timing for insertion and removal of setons, and how does this affect outcomes?** | **12** | **16** | **17** |
| **What is the best strategy for the assessment and monitoring of perianal Crohn’s disease using imaging or other methods?** | **5** | **24** | **10** |
| **What is the best method for closure of the internal opening of a fistula due to perianal Crohn's disease?** | **28** | **8** | **15** |
| **How do diet, nutrition and lifestyle impact on perianal Crohn's disease, and how can they help to prevent or manage the condition?** | **35** | **4** | **8** |
| **How is radiological healing and radiological remission identified and defined?** | **11** | **49** | **33** |
| **What is the best treatment strategy (medical, surgical and combinations) of perianal Crohn’s disease in terms of efficacy, safety and cost-effectiveness, across the full spectrum of the disease?** | **1** | **2** | **1** |
| **What are the best first and subsequent advanced medical therapies (used alone or in combination) for fistulising perianal Crohn’s disease?** | **9** | **7** | **6** |
| **What are the factors which predict severity of perianal Crohn's disease, and what are the early targets for treatment?** | **7** | **18** | **9** |
| **What are the frequency and causes of recurrence of perianal Crohn's disease following treatment, and how can recurrence be reduced?** | **22** | **9** | **12** |
| **What are the impacts of living with perianal Crohn's disease on sexual function, intimacy and relationships, and how is this best managed?** | **17** | **11** | **16** |
| **What are the long-term risks associated with perianal Crohn’s disease, including the risk of cancer and risk to life?** | **32** | **6** | **11** |
| **What are the best strategies for coordinated multi-disciplinary treatment of perianal Crohn's disease?** | **3** | **10** | **5** |
| **What are the impacts of perianal Crohn's disease, and its treatment, on quality of life (QoL) including mental health, daily activities, and social or work life, and how can QoL be improved and patients supported?** | **4** | **1** | **2** |
| **What are the causes and risk factors for perianal Crohn’s disease, and how are they different to the causes and risk factors for luminal Crohn’s disease (bowel inflammation)?** | **2** | **5** | **3** |
| **How can perianal Crohn’s disease be prevented?** | **16** | **3** | **4** |

**Appendix 2.**

| **Longlist of 51 priorities listed in interim prioritisation survey:**   - 1. What are the impacts of perianal Crohn's disease, and its treatment, on quality of life (QoL) including mental health, daily activities, and social or work life, and how can QoL be improved and patients supported?   2. What is the best treatment strategy (medical, surgical and combinations) of perianal Crohn’s disease in terms of efficacy, safety and cost-effectiveness, across the full spectrum of the disease?   3. How can perianal Crohn’s disease be prevented?   4. How do diet, nutrition and lifestyle impact on perianal Crohn's disease, and how can they help to prevent or manage the condition?   5. What are the causes and risk factors for perianal Crohn’s disease, and how are they different to the causes and risk factors for luminal Crohn’s disease (bowel inflammation)?   6. What are the long-term risks associated with perianal Crohn’s disease, including the risk of cancer and risk to life?   7. What are the best first and subsequent advanced medical therapies (used alone or in combination) for fistulising perianal Crohn’s disease?   8. What is the best method for closure of the internal opening of a fistula due to perianal Crohn's disease?   9. What are the frequency and causes of recurrence of perianal Crohn's disease following treatment, and how can recurrence be reduced?   10. What are the best strategies for coordinated multi-disciplinary treatment of perianal Crohn's disease?   11. How can the classification of perianal Crohn's disease be used, and optimised, to guide treatments and predict outcomes?   12. What are the factors which predict severity of perianal Crohn's disease, and what are the early targets for treatment?   13. What is the best strategy for the assessment and monitoring of perianal Crohn’s disease using imaging or other methods?   14. What are the factors that predict treatment response in perianal Crohn's disease, and how can this be used to improve individual outcomes?   15. What are the optimal markers or combination of markers (biological, genetic, imaging, or other) for diagnosis of perianal Crohn's disease, and for stratification of patients with regards to disease course and treatment response (i.e. a biological classification)?   16. What are the impacts of living with perianal Crohn's disease on sexual function, intimacy and relationships, and how is this best managed?   17. How is radiological healing and radiological remission identified and defined?   18. How can the timely diagnosis of perianal Crohn's disease be improved, and which factors contribute to delay?   19. What is the optimal method and timing for insertion and removal of setons, and how does this affect outcomes?   20. How can flares or recurrence of perianal Crohn's disease after treatment be prevented, and what are the risk factors?   21. What are the long-term outcomes of surgical and medical treatments such as biologics or stomas for perianal Crohn's disease?   22. Which conditions overlap with or resemble perianal Crohn's disease, and how can these be identified and treated?   23. What are risk factors for the development of mental health issues in patients with perianal Crohn's disease?   24. Can we identify factors that predict who is likely to develop perianal Crohn’s disease?   25. How can medical and surgical treatments for rectovaginal fistula due to Crohn's disease be improved?   26. How can incontinence and other complications as a result of perianal Crohn's disease, or its treatment, be best managed?   27. What are the main symptoms of perianal Crohn's disease, and when would these prompt investigation?   28. How can perianal Crohn's-related fistula be differentiated from alternate causes of fistula, and what implications does this have for treatment?   29. How can perianal Crohn's disease management be optimised, in the context of Crohn's disease elsewhere in the body (in particular rectal disease)?   30. What are the best care pathways used globally that can promote equitable access to specialist care in perianal Crohn’s disease around the world?   31. How are wounds from surgery for perianal Crohn's disease, and symptoms from the fistulae themselves, best managed or cared for?   32. What is the role of stem cell therapy in perianal Crohn's disease, and which types of patients are suitable?   33. What target drug levels (e.g anti-TNF levels) should we aim for to induce and maintain remission?   34. What are the impacts of perianal Crohn's disease and its treatment on fertility and pregnancy, and how can any risks be managed?   35. When is surgical treatment appropriate for perianal Crohn's disease, and what are the most effective methods with fewest complications?   36. How can the stigma surrounding perianal Crohn's disease be reduced?   37. What is the best way to manage Crohn's disease which affects only the perianal area, and nowhere else in the body?   38. What are the key features of inflammation of the rectum on cross-sectional imaging in perianal Crohn’s disease, and what are the clinical and long-term implications?   39. Can a target for gene therapy be identified for the treatment of perianal Crohn's disease?   40. How can the diagnostic and predictive accuracy of non-invasive imaging techniques and scoring systems in perianal Crohn’s disease be improved, whilst remaining practical?   41. When should patients have a (defunctioning) stoma, and what factors predict the need for stoma formation, and which patients can undergo stoma reversal?   42. What is the role of hyperbaric oxygen therapy in patients with perianal Crohn's disease?   43. Can modified MRE (Magnetic Resonance Enterography) be used to accurately screen, diagnose and assess perianal Crohn's disease?   44. How can digital health interventions (e.g., apps, telemedicine) be utilized to provide ongoing support and improve patient outcomes in perianal Crohn's disease?   45. How does age affect perianal Crohn’s disease?   46. How common is perianal Crohn’s disease globally, and what factors explain the differences across different countries?   47. What proportion of perianal Crohn's disease patients achieve clinical or radiological healing?   48. Can Artificial Intelligence tools be used to help detect malignancy early in perianal Crohn's disease?   49. When is it appropriate to perform examination under anaesthetic, and what approach to assessment is best?   50. How are outcomes for interventions in perianal Crohn's disease best assessed in clinical trials and in real-world data collection?   51. Does standardised reporting in perianal fistula imaging have an impact on patient outcomes? |
| --- |

Appendix 3.

Table – Steering Group composition

| Name | Role/specialty | Region |
| --- | --- | --- |
| Ailsa Hart | PSP Lead (gastroenterology) | UK |
| Phil Tozer | PSP Lead (colorectal surgery) | UK |
| Jonathan Gower | JLA Advisor | JLA, UK |
| Theo Pelly | Co-ordinator | UK |
| Parakkal Deepak | Gastroenterology | USA |
| Jeff McCurdy | Gastroenterology | Canada |
| Shaji Sebastian | Gastroenterology | UK |
| Amir Reza Radmard | Radiology | Iran |
| Phillip Lung | Radiology | UK |
| Jaap Stoker | Radiology | The Netherlands |
| Christianne Buskens | Colorectal Surgery | The Netherlands |
| Lisa Younge | IBD Nurse Consultant | UK |
| Denise Robinson | Clinical Nurse Manager | UK |
| Harjeet Singh | Colorectal Surgery | India |
| Paulo Kotze | Colorectal Surgery | Brazil |
| Yarunessa Khan  Katie Nightingale | Crohn’s and Colitis UK representatives | UK |
| Tom Hough | Patient expert | UK |
| Sarah Markham | Patient expert | UK |
| Anonymous | Patient expert | UK |
| Sameer Mannick | Patient expert | UK |
| Anonymous | Patient expert | India |
| Luke Hanna  Easan Anand  Shivani Joshi  Eathar Shakweh | Data analysis team | UK |

Table – Partner organisations

| Organisation | Type of Organisation |
| --- | --- |
| South Asian IBD Alliance (Global) | Patient |
| United European Gastroenterology (UEG) | Professional |
| Crohn’s and Colitis UK | Patient |
| Treatment Optimisation and Classification of Perianal Crohn’s Disease Consortium (TOpClass) | Professional |
| European Society of Gastrointestinal and Abdominal Radiology | Professional |
| Girls with Guts (USA) | Patient |
| Crohn’s and Colitis Australia | Patient |
| Dukes’ Club (UK) | Professional |
| European Federation of Crohn’s and Colitis Associations | Patient |
| Crohn’s and Colitis Foundation (America) | Patient |

Table – final workshop composition

| Country of final workshop participant | Count (%) | World Bank Income status |
| --- | --- | --- |
| UK | 6 (24%) | High income |
| Australia | 5 (20%) | High income |
| USA | 3 (12%) | High income |
| India | 2 (8%) | Lower-middle-income |
| Netherlands | 2 (8%) | High-income |
| Canada | 2 (8%) | High-income |
| Belgium | 2 (8%) | High-income |
| United Arab Emirates | 1 (4%) | High-income |
| Iran | 1 (4%) | Upper-middle-income |
| New Zealand | 1 (4%) | High-income |
| Portugal | 1 (4%) | High-income |
